# Supplementary material for: Improved sequence mapping using a complete reference genome and lift-over
Source: Nat Methods. Author manuscript; Available in PMC 2024 Dec 2. (PMC11610747; doi:10.1038/s41592-023-02069-6)
Supplement: Supplementary material — Figure S1: Regions unique to T2T-CHM131 compared to GRCh38 (blue) and high quality calls from DeepVariant in these regions (red). Figure S2: Mapping accuracy using simulated reads that carry GRCh38-based HG001 genotypes2. Figure S3: Peak memory usage of levioSAM2 and direct-to-GRC pipelines using a real 30× WGS dataset from HG002. Figure S4: Thread scaling of levioSAM2-lift. 3.6M pairs (0.3 × coverage) of real Illumina reads from the real HG002 dataset were used. Wall clock time (second) and peak memory usage (MB) were measured using GNU Time. Figure S5: Small variant calling performance in difficult regions. a, Small variant calling accuracy in major difficult genomic regions for HG002. b, GIAB stratified regions with top small variant calling error reduction densities by levioSAM2. Small variants in both plots were called using using DeepVariant. Figure S6: IGV visualization near chr5:21,543,010. The reads were grouped using the allele at chr5:21,543,010. A 174-bp DEL was called when using direct-to-GRCh37, matching the GIAB Tier 1 SV callset. However, personalized whole-genome assemblies suggested collapse mapping in this region and the CHM13-to-GRCh37 mappings showed better concordance with the assemblies. Figure S7: IGV visualization near chr7:61,880,665. The reads were grouped using the allele at chr7:61,880,665. A 166-bp DEL was called when using direct-to-GRCh37, matching the GIAB Tier 1 SV callset. However, personalized whole-genome assemblies suggested collapse mapping in this region and the CHM13-to-GRCh37 mappings showed better concordance with the assemblies. Figure S8: DeepVariant calls in chr7:152,104,140-152,104,343 (located in the KMT2C gene). This is a region annotated as high confidence (“Confident regions”) but has no truth variants (“HG002 truth variants”). Gray bars in the “DeepVariant calls” track show homozygous reference variant calls. Colors other than gray in the “GRCh37” and “GRCh37 coverage” tracks show alternate alleles. Table S1: Softw [file NIHMS2035437-supplement-Supplementary_material.pdf]

bioRxiv preprint doi: https://doi.org/10.1101/2022.04.27.489683; this version posted April 28, 2022. The copyright holder for this preprint (which was not certified by peer review) is the author/funder, who has granted bioRxiv a license to display the preprint in perpetuity. It is made available under aCC-BY 4.0 International license.

Supplementary Notes

S1 DeepVariant calls in difficult-to-map regions

We examined the DeepVariant calls in the *KMT2C* gene, where there were known mapping collapses for HG002 data when using direct-to-GRCh37 (see Results). We noticed that DeepVariant made many homozygous reference calls even when the variant allele fraction (VAF) were as high as 0.82 (Figure S8). The GIAB truth set reported few variants in this region. We reasoned that DeepVariant could "recognize" mapping artifacts and adjust its decisions in difficult-to-map regions.

S2 Computational efficiency of levioSAM2

We measured the CPU time and peak memory usage of each step in the levioSAM2 and typical pipelines (Figure 6b and Figure S3). In the levioSAM2 workflows, lifting alignments over took 19.2% CPU time (7.1 hours) for CHM13-to-GRCh37 and 13.6% (6.5 hours) for CHM13-to-GRCh38. The majority of the CPU-time usage for the levioSAM2 workflow was in the remapping step, taking 66.2% (24.5 hours; GRCh37) and 74.6% (35.6 hours; GRCh38) of time. Mapping the deferred reads took longer and had a higher memory footprint compared to the direct-to-target mapping task, likely because of the higher incidence of repetitive alignments for deferred reads. The most memory-consuming step was levioSAM2-collate, since we used a hash map to store unpaired deferred reads. For memory-limited systems, it will be straightforward to reduce the memory bottleneck with a marginal increase in CPU time .

bioRxiv preprint doi: https://doi.org/10.1101/2022.04.27.489683; this version posted April 28, 2022. The copyright holder for this preprint (which was not certified by peer review) is the author/funder, who has granted bioRxiv a license to display the preprint in perpetuity. It is made available under aCC-BY 4.0 International license.

Supplementary Figures

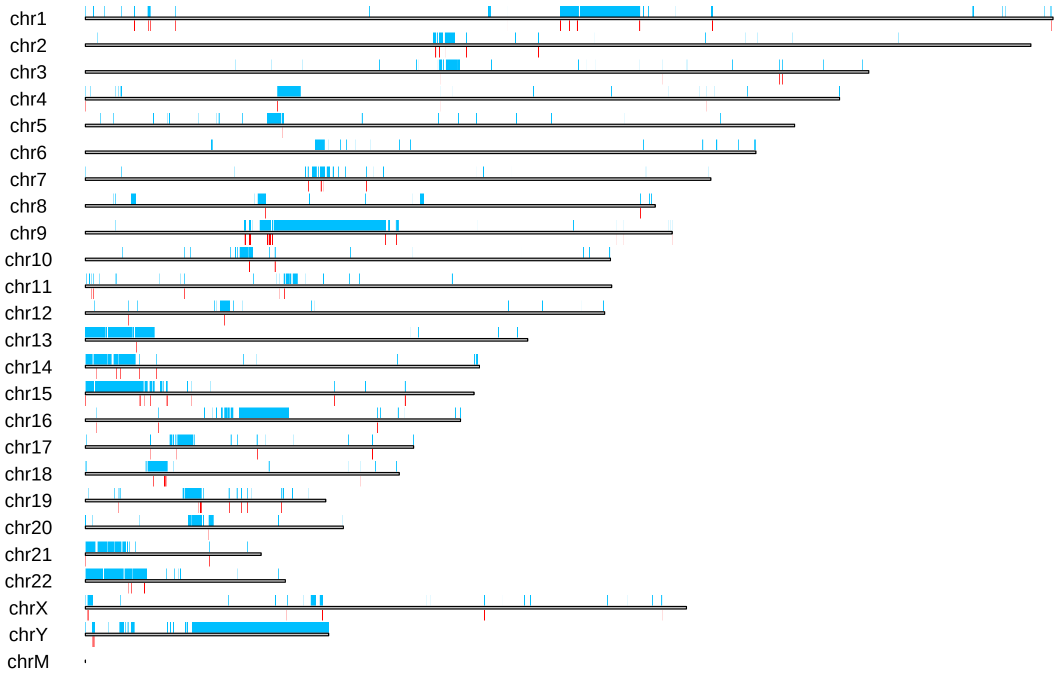

Figure S1: Regions unique to T2T-CHM13[1] compared to GRCh38 (blue) and high quality calls from DeepVariant in these regions (red).

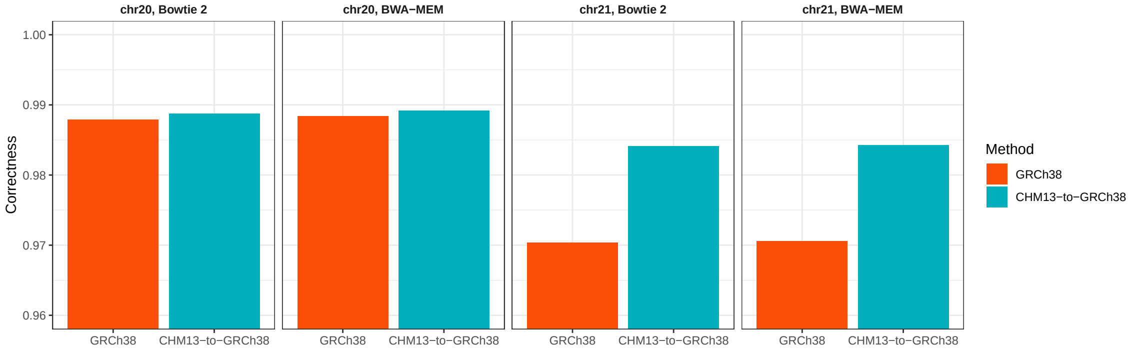

Figure S2: Mapping accuracy using simulated reads that carry GRCh38-based HG001 genotypes[2].

bioRxiv preprint doi: https://doi.org/10.1101/2022.04.27.489683; this version posted April 28, 2022. The copyright holder for this preprint (which was not certified by peer review) is the author/funder, who has granted bioRxiv a license to display the preprint in perpetuity. It is made available under aCC-BY 4.0 International license.

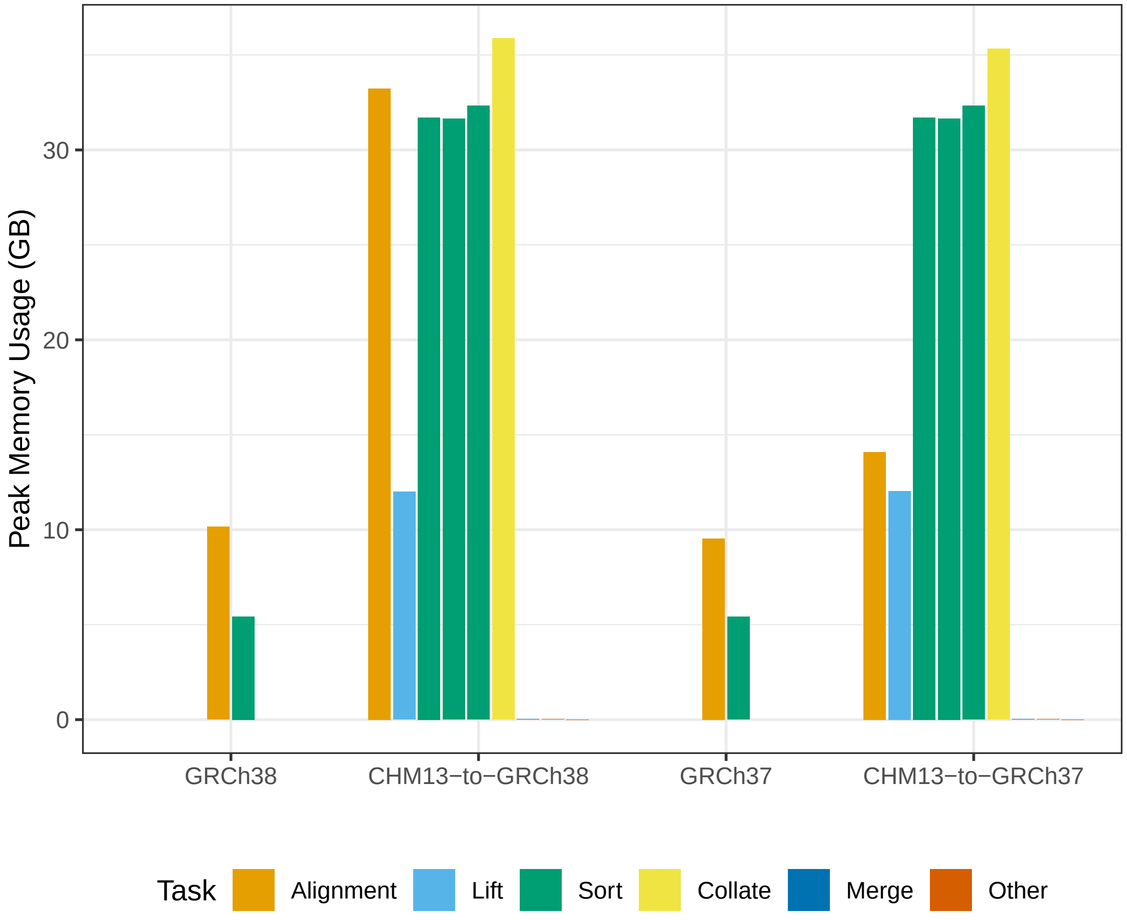

Figure S3: Peak memory usage of levioSAM2 and direct-to-GRC pipelines using a real 30× WGS dataset from HG002.

bioRxiv preprint doi: https://doi.org/10.1101/2022.04.27.489683; this version posted April 28, 2022. The copyright holder for this preprint (which was not certified by peer review) is the author/funder, who has granted bioRxiv a license to display the preprint in perpetuity. It is made available under aCC-BY 4.0 International license.

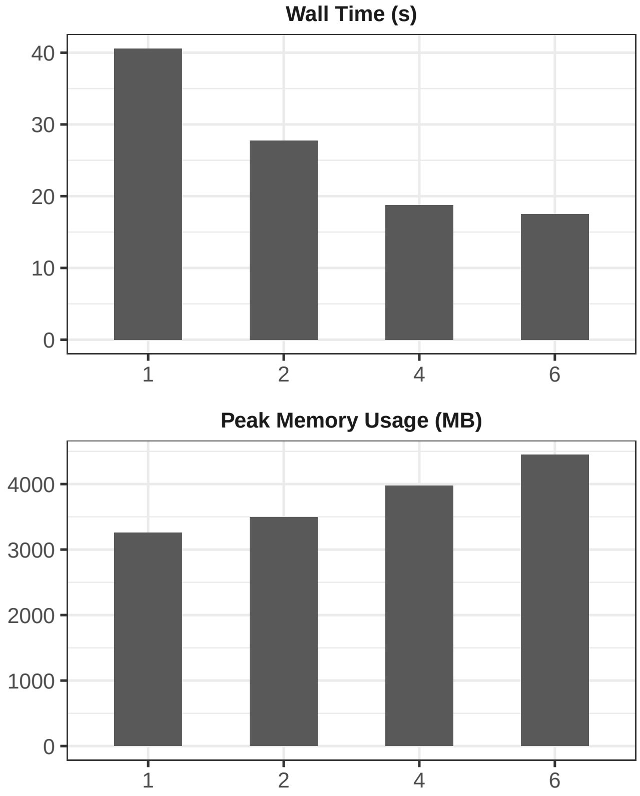

Figure S4: Thread scaling of levioSAM2-lift. 3.6M pairs (0.3 $\times$ coverage) of real Illumina reads from the real HG002 dataset were used. Wall clock time (second) and peak memory usage (MB) were measured using GNU Time.

bioRxiv preprint doi: https://doi.org/10.1101/2022.04.27.489683; this version posted April 28, 2022. The copyright holder for this preprint (which was not certified by peer review) is the author/funder, who has granted bioRxiv a license to display the preprint in perpetuity. It is made available under aCC-BY 4.0 International license.

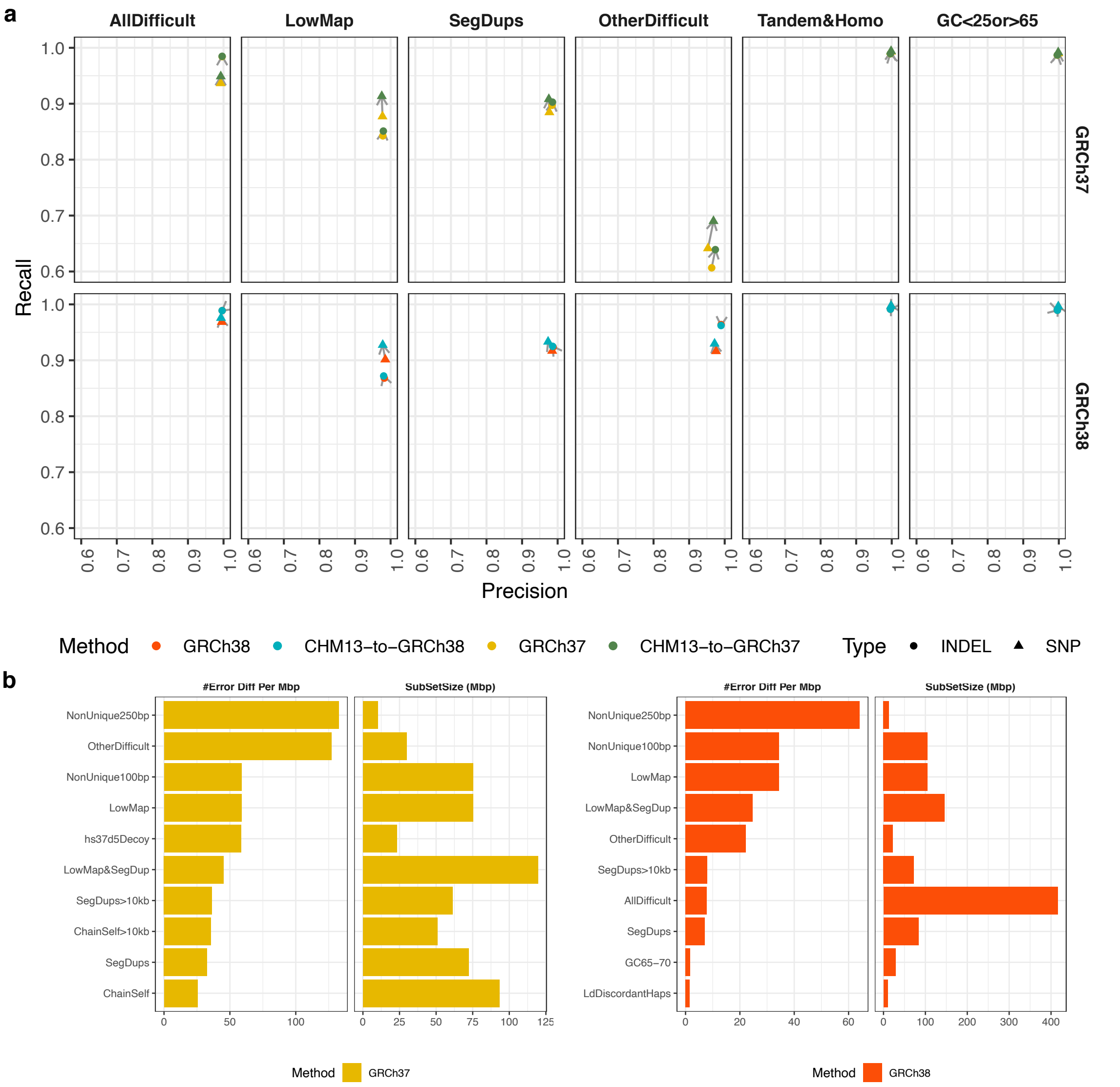

Figure S5: Small variant calling performance in difficult regions. a, Small variant calling accuracy in major difficult genomic regions for HG002. **b**, GIAB stratified regions with top small variant calling error reduction densities by levioSAM2. Small variants in both plots were called using using DeepVariant.

bioRxiv preprint doi: https://doi.org/10.1101/2022.04.27.489683; this version posted April 28, 2022. The copyright holder for this preprint (which was not certified by peer review) is the author/funder, who has granted bioRxiv a license to display the preprint in perpetuity. It is made available under aCC-BY 4.0 International license.

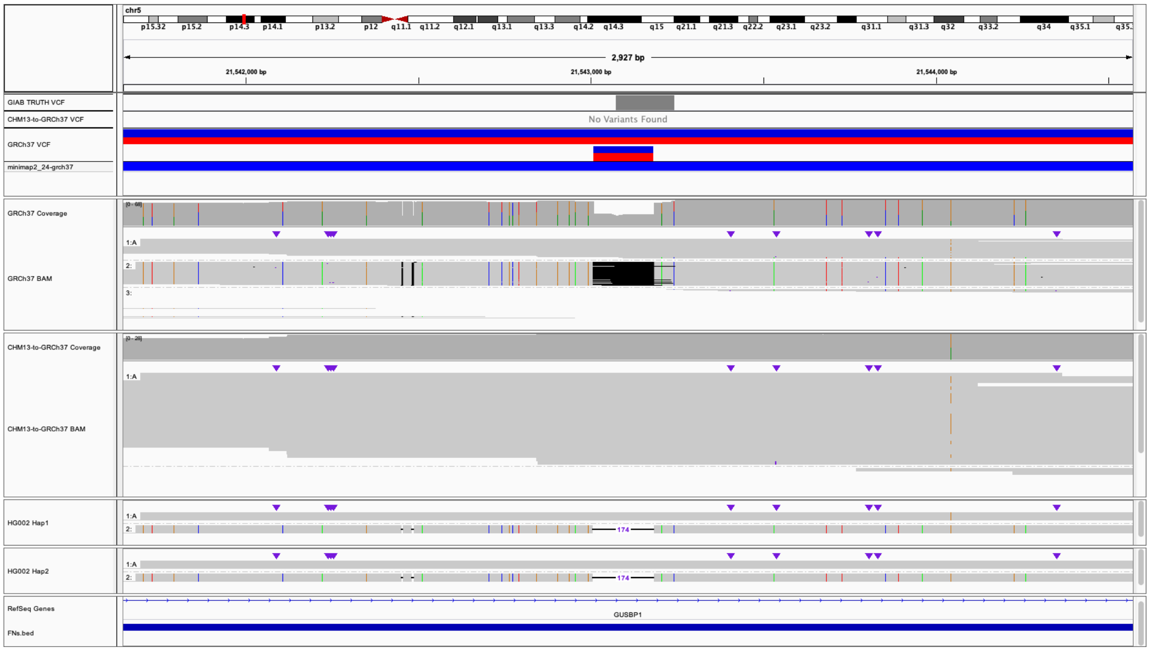

Figure S6: IGV visualization near chr5:21,543,010. The reads were grouped using the allele at chr5:21,543,010. A 174-bp DEL was called when using direct-to-GRCh37, matching the GIAB Tier 1 SV callset. However, personalized whole-genome assemblies suggested collapse mapping in this region and the CHM13-to-GRCh37 mappings showed better concordance with the assemblies.

bioRxiv preprint doi: https://doi.org/10.1101/2022.04.27.489683; this version posted April 28, 2022. The copyright holder for this preprint (which was not certified by peer review) is the author/funder, who has granted bioRxiv a license to display the preprint in perpetuity. It is made available under aCC-BY 4.0 International license.

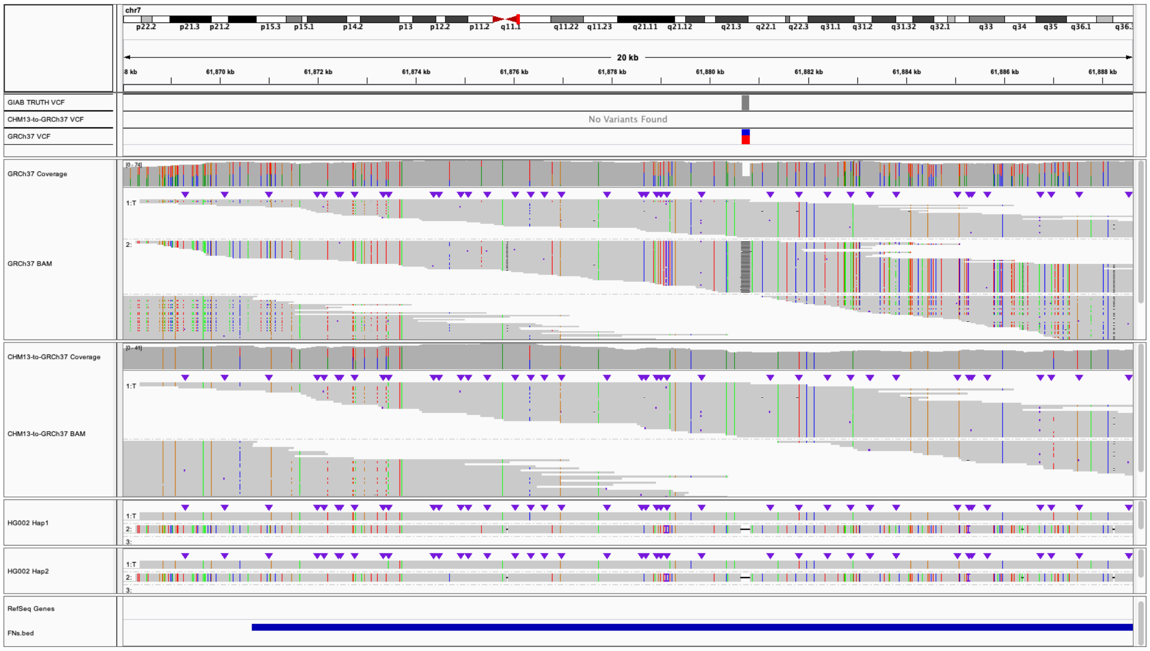

Figure S7: IGV visualization near chr7:61,880,665. The reads were grouped using the allele at chr7:61,880,665. A 166-bp DEL was called when using direct-to-GRCh37, matching the GIAB Tier 1 SV callset. However, personalized whole-genome assemblies suggested collapse mapping in this region and the CHM13-to-GRCh37 mappings showed better concordance with the assemblies.

bioRxiv preprint doi: https://doi.org/10.1101/2022.04.27.489683; this version posted April 28, 2022. The copyright holder for this preprint (which was not certified by peer review) is the author/funder, who has granted bioRxiv a license to display the preprint in perpetuity. It is made available under aCC-BY 4.0 International license.

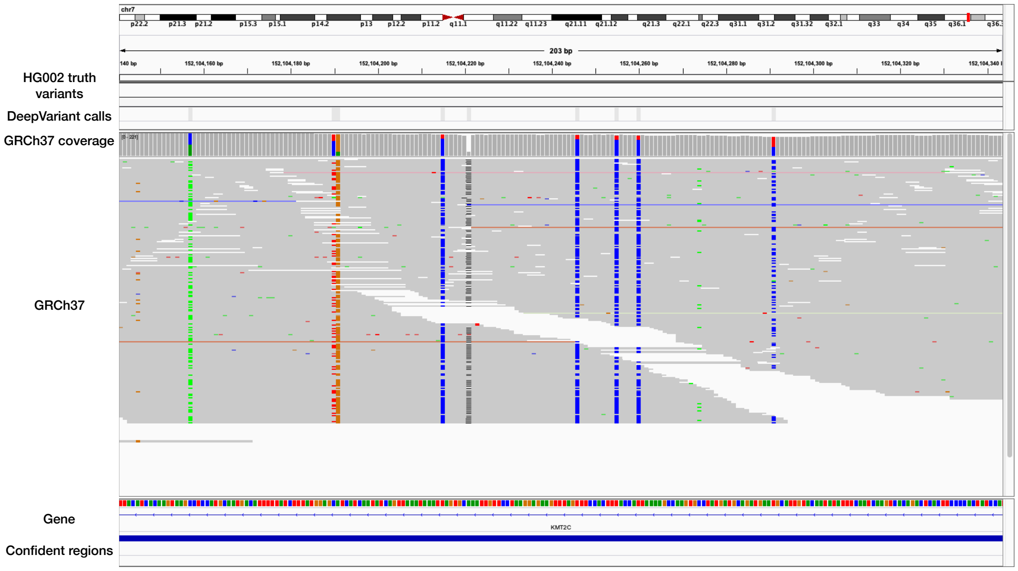

Figure S8: DeepVariant calls in chr7:152,104,140-152,104,343 (located in the *KMT2C* gene). This is a region annotated as high confidence ("Confident regions") but has no truth variants ("HG002 truth variants"). Gray bars in the "DeepVariant calls" track show homozygous reference variant calls. Colors other than gray in the "GRCh37" and "GRCh37 coverage" tracks show alternate alleles.

bioRxiv preprint doi: https://doi.org/10.1101/2022.04.27.489683; this version posted April 28, 2022. The copyright holder for this preprint (which was not certified by peer review) is the author/funder, who has granted bioRxiv a license to display the preprint in perpetuity. It is made available under aCC-BY 4.0 International license.

Supplementary Tables

Table S1: Software version

levioSAM2	v0.2.0
BWA-MEM[3]	0.7.17-r1188
minimap2[4]	2.24-r1122
Winnowmap2[5]	2.03
Bowtie 2[6]	2.3.5.1
bedtools[7]	v2.30.0-48-g868a9a24
GATK[8]	v4.2.2.0
HTSJDK[9]	2.24.1
Picard[10]	2.25.4
DeepVariant[11]	1.2.0
Hap.py[12]	v0.3.8-17-gf15de4a
Sniffles2[13]	2.0.1
whatshap[14]	1.2.1
truvari[15]	v2.1
nf-LO[16]	1.5.1
liftOver[17]	*accessed on Sep 14, 2021*
CrossMap[18]	v0.5.4
mason2[19]	2.0.9
GNU Time[20]	1.9
IGV[21]	2.6.3

bioRxiv preprint doi: https://doi.org/10.1101/2022.04.27.489683; this version posted April 28, 2022. The copyright holder for this preprint (which was not certified by peer review) is the author/funder, who has granted bioRxiv a license to display the preprint in perpetuity. It is made available under aCC-BY 4.0 International license.

Table S2: Small variant calling accuracy for 30× WGS datasets using BWA-MEM–GATK-HaplotypeCaller in all GIAB v4.2.1 regions[22]

Sample	Method	Type	Recall	Precision	F_1	TP	FN	FP
HG001	GRCh38	SNP	0.9932	0.9875	0.9903	3232144	22228	41050
HG001	CHM13-to-GRCh38	SNP	0.9950	0.9908	0.9929	3238118	16254	30230
HG001	GRCh37	SNP	0.9869	0.9790	0.9829	3209109	42734	68868
HG001	CHM13-to-GRCh37	SNP	0.9900	0.9903	0.9901	3219275	32568	31572
HG001	GRCh38	INDEL	0.9902	0.9889	0.9895	463115	4578	5418
HG001	CHM13-to-GRCh38	INDEL	0.9904	0.9907	0.9905	463195	4498	4537
HG001	GRCh37	INDEL	0.9863	0.9832	0.9848	460484	6391	8148
HG001	CHM13-to-GRCh37	INDEL	0.9870	0.9897	0.9884	460816	6059	4981
HG002	GRCh38	SNP	0.9922	0.9877	0.9899	3338776	26350	41484
HG002	CHM13-to-GRCh38	SNP	0.9943	0.9912	0.9927	3345806	19320	29656
HG002	GRCh37	SNP	0.9859	0.9802	0.9831	3305380	47305	66646
HG002	CHM13-to-GRCh37	SNP	0.9890	0.9909	0.9900	3315802	36883	30406
HG002	GRCh38	INDEL	0.9893	0.9889	0.9891	519842	5625	6059
HG002	CHM13-to-GRCh38	INDEL	0.9895	0.9911	0.9903	519929	5538	4887
HG002	GRCh37	INDEL	0.9858	0.9843	0.9851	514968	7421	8551
HG002	CHM13-to-GRCh37	INDEL	0.9864	0.9903	0.9884	515300	7089	5244
HG005	GRCh38	SNP	0.9914	0.9876	0.9895	3247494	28120	40927
HG005	CHM13-to-GRCh38	SNP	0.9936	0.9903	0.9920	3254520	21094	31718
HG005	GRCh37	SNP	0.9861	0.9801	0.9831	3220745	45244	65350
HG005	CHM13-to-GRCh37	SNP	0.9894	0.9904	0.9899	3231517	34472	31338
HG005	GRCh38	INDEL	0.9922	0.9900	0.9911	413504	3263	4309
HG005	CHM13-to-GRCh38	INDEL	0.9923	0.9917	0.9920	413577	3190	3566
HG005	GRCh37	INDEL	0.9883	0.9844	0.9864	408990	4834	6685
HG005	CHM13-to-GRCh37	INDEL	0.9891	0.9912	0.9902	409333	4491	3763

bioRxiv preprint doi: https://doi.org/10.1101/2022.04.27.489683; this version posted April 28, 2022. The copyright holder for this preprint (which was not certified by peer review) is the author/funder, who has granted bioRxiv a license to display the preprint in perpetuity. It is made available under aCC-BY 4.0 International license.

Table S3: Small variant calling accuracy for 30× WGS datasets using BWA-MEM–DeepVariant in all GIAB v4.2.1 regions[22]

Sample	Method	Type	Recall	Precision	F_1	TP	FN	FP
HG001	GRCh38	SNP	0.9945	0.9983	0.9964	3236509	17863	5417
HG001	CHM13-to-GRCh38	SNP	0.9958	0.9981	0.9970	3240823	13549	6144
HG001	GRCh37	SNP	0.9883	0.9978	0.9931	3213802	38041	6937
HG001	CHM13-to-GRCh37	SNP	0.9908	0.9980	0.9944	3222057	29786	6364
HG001	GRCh38	INDEL	0.9925	0.9967	0.9946	464179	3514	1609
HG001	CHM13-to-GRCh38	INDEL	0.9925	0.9966	0.9946	464176	3517	1626
HG001	GRCh37	INDEL	0.9888	0.9966	0.9927	461638	5237	1637
HG001	CHM13-to-GRCh37	INDEL	0.9893	0.9966	0.9930	461883	4992	1623
HG002	GRCh38	SNP	0.9937	0.9990	0.9963	3343863	21263	3436
HG002	CHM13-to-GRCh38	SNP	0.9951	0.9985	0.9968	3348636	16490	4881
HG002	GRCh37	SNP	0.9874	0.9985	0.9929	3310312	42373	5059
HG002	CHM13-to-GRCh37	SNP	0.9898	0.9985	0.9941	3318525	34160	4935
HG002	GRCh38	INDEL	0.9919	0.9972	0.9946	521223	4244	1506
HG002	CHM13-to-GRCh38	INDEL	0.9919	0.9972	0.9945	521209	4258	1548
HG002	GRCh37	INDEL	0.9884	0.9971	0.9927	516310	6079	1560
HG002	CHM13-to-GRCh37	INDEL	0.9888	0.9971	0.9929	516527	5862	1561
HG005	GRCh38	SNP	0.9930	0.9986	0.9958	3252742	22872	4588
HG005	CHM13-to-GRCh38	SNP	0.9945	0.9982	0.9963	3257465	18149	5812
HG005	GRCh37	SNP	0.9878	0.9984	0.9931	3226259	39730	5227
HG005	CHM13-to-GRCh37	SNP	0.9904	0.9984	0.9944	3234785	31204	5309
HG005	GRCh38	INDEL	0.9929	0.9977	0.9953	413827	2941	993
HG005	CHM13-to-GRCh38	INDEL	0.9930	0.9976	0.9953	413845	2923	1021
HG005	GRCh37	INDEL	0.9892	0.9977	0.9934	409353	4472	969
HG005	CHM13-to-GRCh37	INDEL	0.9898	0.9977	0.9937	409620	4205	987

Table S4: Small variant calling accuracy for 30× WGS datasets using BWA-MEM–GATK-HaplotypeCaller in GIAB CMRG regions for HG002[23]

Method	Type	Recall	Precision	F_1	TP	FN	FP
GRCh38	SNP	0.9569	0.9483	0.9526	16824	758	911
CHM13-to-GRCh38	SNP	0.9652	0.9758	0.9705	16971	611	418
GRCh37	SNP	0.9481	0.9036	0.9253	16978	929	1798
CHM13-to-GRCh37	SNP	0.9521	0.9677	0.9598	17049	858	565
GRCh38	INDEL	0.9387	0.9520	0.9453	3398	222	184
CHM13-to-GRCh38	INDEL	0.9412	0.9726	0.9566	3407	213	103
GRCh37	INDEL	0.9251	0.9155	0.9202	3382	274	336
CHM13-to-GRCh37	INDEL	0.9275	0.9643	0.9456	3391	265	135

bioRxiv preprint doi: https://doi.org/10.1101/2022.04.27.489683; this version posted April 28, 2022. The copyright holder for this preprint (which was not certified by peer review) is the author/funder, who has granted bioRxiv a license to display the preprint in perpetuity. It is made available under aCC-BY 4.0 International license.

Table S5: Small variant calling accuracy for 30× WGS datasets using BWA-MEM–DeepVariant in GIAB CMRG regions for HG002[23]

Method	Type	Recall	Precision	F_1	TP	FN	FP
GRCh38	SNP	0.9616	0.9854	0.9734	16907	675	250
CHM13-to-GRCh38	SNP	0.9700	0.9904	0.9801	17055	527	164
GRCh37	SNP	0.9547	0.9790	0.9667	17095	812	366
CHM13-to-GRCh37	SNP	0.9591	0.9896	0.9741	17175	732	179
GRCh38	INDEL	0.9340	0.9735	0.9533	3381	239	98
CHM13-to-GRCh38	INDEL	0.9362	0.9762	0.9558	3389	231	88
GRCh37	INDEL	0.9248	0.9667	0.9453	3381	275	124
CHM13-to-GRCh37	INDEL	0.9267	0.9751	0.9503	3388	268	92

Table S6: Difficult regions stratified by GIAB[23]. The sizes are calculated after intersecting stratified regions with the GIAB v4.2.1 confident regions for HG002[22]

Reference	GIAB subset name	Legend	Size
GRCh38	alldifficultregions	AllDifficult	415,601,891
GRCh38	gclt25orgt65_slop50	ExtremeGC	164,164,687
GRCh38	AllTandemRepeatsandHomopolymers_slop5	Tandem&Homo	121,406,218
GRCh38	lowmappabilityall	LowMap	105,002,312
GRCh38	segdups	SegDups	83,843,041
GRCh38	allOtherDifficultregions	OtherDifficult	22,511,200
GRCh37	alldifficultregions	AllDifficult	393,128,724
GRCh37	AllTandemRepeatsandHomopolymers_slop5	Tandem&Homo	121,049,426
GRCh37	lowmappabilityall	LowMap	75,276,894
GRCh37	segdups	SegDups	72,412,437
GRCh37	gclt25orgt65_slop50	ExtremeGC	62,521,603
GRCh37	allOtherDifficultregions	OtherDifficult	29,884,114

bioRxiv preprint doi: https://doi.org/10.1101/2022.04.27.489683; this version posted April 28, 2022. The copyright holder for this preprint (which was not certified by peer review) is the author/funder, who has granted bioRxiv a license to display the preprint in perpetuity. It is made available under aCC-BY 4.0 International license.

Table S7: Small variant calling accuracy for 30× WGS datasets using BWA-MEM–GATK-HaplotypeCaller in GIAB difficult regions for HG002[23]

Method	Type	Subset	Recall	Precision	F_1	TP	FN	FP
GRCh38	SNP	GC<25or>65	0.9943	0.9834	0.9889	212359	1209	3576
CHM13-to-GRCh38	SNP	GC<25or>65	0.9954	0.9884	0.9919	212591	977	2487
GRCh37	SNP	GC<25or>65	0.9891	0.9759	0.9825	210064	2318	5179
CHM13-to-GRCh37	SNP	GC<25or>65	0.9910	0.9882	0.9896	210470	1912	2514
GRCh38	SNP	Tandem&Homo	0.9948	0.9836	0.9891	181945	960	3059
CHM13-to-GRCh38	SNP	Tandem&Homo	0.9951	0.9862	0.9906	182017	888	2564
GRCh37	SNP	Tandem&Homo	0.9909	0.9795	0.9852	180733	1662	3808
CHM13-to-GRCh37	SNP	Tandem&Homo	0.9919	0.9872	0.9896	180925	1470	2357
GRCh38	SNP	OtherDifficult	0.8941	0.6999	0.7852	49090	5814	20974
CHM13-to-GRCh38	SNP	OtherDifficult	0.9148	0.8232	0.8666	50224	4680	10750
GRCh37	SNP	OtherDifficult	0.6308	0.5328	0.5777	41407	24230	36307
CHM13-to-GRCh37	SNP	OtherDifficult	0.6828	0.8073	0.7398	44814	20823	10694
GRCh38	SNP	SegDups	0.9076	0.8370	0.8709	109771	11177	21381
CHM13-to-GRCh38	SNP	SegDups	0.9302	0.8819	0.9054	112508	8440	15071
GRCh37	SNP	SegDups	0.8761	0.7342	0.7989	96344	13627	34874
CHM13-to-GRCh37	SNP	SegDups	0.9043	0.8719	0.8878	99445	10526	14616
GRCh38	SNP	LowMap	0.8806	0.8918	0.8861	169632	23008	20590
CHM13-to-GRCh38	SNP	LowMap	0.9171	0.9241	0.9206	176665	15975	14517
GRCh37	SNP	LowMap	0.8516	0.8142	0.8325	112749	19652	25729
CHM13-to-GRCh37	SNP	LowMap	0.9006	0.9206	0.9105	119240	13161	10282
GRCh38	SNP	AllDifficult	0.9611	0.9482	0.9546	618558	25007	33821
CHM13-to-GRCh38	SNP	AllDifficult	0.9718	0.9647	0.9682	625442	18123	22947
GRCh37	SNP	AllDifficult	0.9296	0.9084	0.9189	556892	42168	56269
CHM13-to-GRCh37	SNP	AllDifficult	0.9450	0.9597	0.9523	566140	32920	23803
GRCh38	INDEL	GC<25or>65	0.9866	0.9869	0.9868	49835	675	680
CHM13-to-GRCh38	INDEL	GC<25or>65	0.9870	0.9894	0.9882	49854	656	549
GRCh37	INDEL	GC<25or>65	0.9840	0.9815	0.9828	49672	806	965
CHM13-to-GRCh37	INDEL	GC<25or>65	0.9847	0.9887	0.9867	49706	772	587
GRCh38	INDEL	Tandem&Homo	0.9881	0.9918	0.9899	334899	4042	2941
CHM13-to-GRCh38	INDEL	Tandem&Homo	0.9882	0.9923	0.9902	334933	4008	2782
GRCh37	INDEL	Tandem&Homo	0.9861	0.9909	0.9885	331136	4665	3242
CHM13-to-GRCh37	INDEL	Tandem&Homo	0.9865	0.9923	0.9894	331274	4527	2739
GRCh38	INDEL	OtherDifficult	0.9573	0.8609	0.9065	10227	456	1762
CHM13-to-GRCh38	INDEL	OtherDifficult	0.9591	0.9285	0.9436	10246	437	840
GRCh37	INDEL	OtherDifficult	0.5957	0.4563	0.5168	2803	1902	3370
CHM13-to-GRCh37	INDEL	OtherDifficult	0.6344	0.7358	0.6814	2985	1720	1085

Continued on next page

bioRxiv preprint doi: https://doi.org/10.1101/2022.04.27.489683; this version posted April 28, 2022. The copyright holder for this preprint (which was not certified by peer review) is the author/funder, who has granted bioRxiv a license to display the preprint in perpetuity. It is made available under aCC-BY 4.0 International license.

Table S7 – *Continued from previous page*

Method	Type	Subset	Recall	Precision	F_1	TP	FN	FP
GRCh38	INDEL	SegDups	0.9137	0.8599	0.8860	9888	934	1649
CHM13-to-GRCh38	INDEL	SegDups	0.9168	0.9037	0.9102	9922	900	1082
GRCh37	INDEL	SegDups	0.8876	0.7522	0.8143	8809	1115	2966
CHM13-to-GRCh37	INDEL	SegDups	0.8951	0.8851	0.8901	8883	1041	1179
GRCh38	INDEL	LowMap	0.8486	0.8678	0.8581	8836	1576	1362
CHM13-to-GRCh38	INDEL	LowMap	0.8552	0.9045	0.8792	8904	1508	951
GRCh37	INDEL	LowMap	0.8211	0.7691	0.7943	5917	1289	1798
CHM13-to-GRCh37	INDEL	LowMap	0.8333	0.8941	0.8626	6005	1201	720
GRCh38	INDEL	AllDifficult	0.9853	0.9870	0.9862	365008	5440	5096
CHM13-to-GRCh38	INDEL	AllDifficult	0.9855	0.9896	0.9876	365091	5357	4051
GRCh37	INDEL	AllDifficult	0.9808	0.9814	0.9811	357465	7014	7193
CHM13-to-GRCh37	INDEL	AllDifficult	0.9816	0.9887	0.9851	357770	6709	4349

bioRxiv preprint doi: https://doi.org/10.1101/2022.04.27.489683; this version posted April 28, 2022. The copyright holder for this preprint (which was not certified by peer review) is the author/funder, who has granted bioRxiv a license to display the preprint in perpetuity. It is made available under aCC-BY 4.0 International license.

Table S8: Small variant calling accuracy for 30× WGS datasets using BWA-MEM–DeepVariant in GIAB difficult regions for HG002[23]

Method	Type	Subset	Recall	Precision	F_1	TP	FN	FP
GRCh38	SNP	GC<25or>65	0.9957	0.9990	0.9973	212648	920	218
CHM13-to-GRCh38	SNP	GC<25or>65	0.9963	0.9985	0.9974	212779	789	318
GRCh37	SNP	GC<25or>65	0.9901	0.9983	0.9942	210285	2097	359
CHM13-to-GRCh37	SNP	GC<25or>65	0.9919	0.9984	0.9951	210657	1725	342
GRCh38	SNP	Tandem&Homo	0.9972	0.9983	0.9977	182389	516	310
CHM13-to-GRCh38	SNP	Tandem&Homo	0.9974	0.9982	0.9978	182423	482	340
GRCh37	SNP	Tandem&Homo	0.9932	0.9980	0.9956	181146	1249	363
CHM13-to-GRCh37	SNP	Tandem&Homo	0.9941	0.9981	0.9961	181311	1084	346
GRCh38	SNP	OtherDifficult	0.9163	0.9757	0.9450	50306	4598	1256
CHM13-to-GRCh38	SNP	OtherDifficult	0.9297	0.9715	0.9502	51045	3859	1497
GRCh37	SNP	OtherDifficult	0.6414	0.9530	0.7668	42101	23536	2076
CHM13-to-GRCh37	SNP	OtherDifficult	0.6901	0.9685	0.8059	45294	20343	1474
GRCh38	SNP	SegDups	0.9169	0.9847	0.9496	110896	10052	1729
CHM13-to-GRCh38	SNP	SegDups	0.9332	0.9731	0.9528	112872	8076	3117
GRCh37	SNP	SegDups	0.8847	0.9768	0.9284	97286	12685	2316
CHM13-to-GRCh37	SNP	SegDups	0.9082	0.9752	0.9405	99877	10094	2537
GRCh38	SNP	LowMap	0.9014	0.9851	0.9414	173653	18987	2634
CHM13-to-GRCh38	SNP	LowMap	0.9275	0.9779	0.9520	178675	13965	4038
GRCh37	SNP	LowMap	0.8773	0.9772	0.9246	116156	16245	2705
CHM13-to-GRCh37	SNP	LowMap	0.9136	0.9752	0.9434	120959	11442	3077
GRCh38	SNP	AllDifficult	0.9685	0.9951	0.9816	623274	20291	3073
CHM13-to-GRCh38	SNP	AllDifficult	0.9758	0.9929	0.9842	627965	15600	4517
GRCh37	SNP	AllDifficult	0.9366	0.9920	0.9635	561083	37977	4534
CHM13-to-GRCh37	SNP	AllDifficult	0.9489	0.9922	0.9701	568432	30628	4467
GRCh38	INDEL	GC<25or>65	0.9895	0.9959	0.9927	49979	531	211
CHM13-to-GRCh38	INDEL	GC<25or>65	0.9895	0.9960	0.9927	49978	532	205
GRCh37	INDEL	GC<25or>65	0.9869	0.9958	0.9913	49815	663	216
CHM13-to-GRCh37	INDEL	GC<25or>65	0.9871	0.9959	0.9915	49826	652	213
GRCh38	INDEL	Tandem&Homo	0.9914	0.9962	0.9938	336023	2918	1360
CHM13-to-GRCh38	INDEL	Tandem&Homo	0.9913	0.9961	0.9937	336001	2940	1385
GRCh37	INDEL	Tandem&Homo	0.9894	0.9962	0.9928	332240	3561	1346
CHM13-to-GRCh37	INDEL	Tandem&Homo	0.9896	0.9962	0.9928	332294	3507	1366
GRCh38	INDEL	OtherDifficult	0.9639	0.9899	0.9767	10299	386	110
CHM13-to-GRCh38	INDEL	OtherDifficult	0.9622	0.9897	0.9758	10281	404	112
GRCh37	INDEL	OtherDifficult	0.6066	0.9634	0.7445	2854	1851	109
CHM13-to-GRCh37	INDEL	OtherDifficult	0.6391	0.9737	0.7717	3007	1698	82

Continued on next page

bioRxiv preprint doi: https://doi.org/10.1101/2022.04.27.489683; this version posted April 28, 2022. The copyright holder for this preprint (which was not certified by peer review) is the author/funder, who has granted bioRxiv a license to display the preprint in perpetuity. It is made available under aCC-BY 4.0 International license.

Table S8 – *Continued from previous page*

Method	Type	Subset	Recall	Precision	F_1	TP	FN	FP
GRCh38	INDEL	SegDups	0.9236	0.9874	0.9544	9995	827	130
CHM13-to-GRCh38	INDEL	SegDups	0.9250	0.9859	0.9545	10010	812	146
GRCh37	INDEL	SegDups	0.8972	0.9845	0.9388	8904	1020	143
CHM13-to-GRCh37	INDEL	SegDups	0.9029	0.9854	0.9423	8960	964	135
GRCh38	INDEL	LowMap	0.8681	0.9825	0.9218	9040	1373	163
CHM13-to-GRCh38	INDEL	LowMap	0.8720	0.9805	0.9231	9080	1333	182
GRCh37	INDEL	LowMap	0.8425	0.9782	0.9053	6073	1135	137
CHM13-to-GRCh37	INDEL	LowMap	0.8511	0.9796	0.9109	6134	1073	129
GRCh38	INDEL	AllDifficult	0.9889	0.9962	0.9925	366326	4122	1470
CHM13-to-GRCh38	INDEL	AllDifficult	0.9888	0.9961	0.9925	366308	4140	1509
GRCh37	INDEL	AllDifficult	0.9842	0.9960	0.9901	358734	5746	1517
CHM13-to-GRCh37	INDEL	AllDifficult	0.9848	0.9960	0.9904	358931	5549	1519

bioRxiv preprint doi: https://doi.org/10.1101/2022.04.27.489683; this version posted April 28, 2022. The copyright holder for this preprint (which was not certified by peer review) is the author/funder, who has granted bioRxiv a license to display the preprint in perpetuity. It is made available under aCC-BY 4.0 International license.

Table S9: Small variant calling accuracy for 28× WGS PacBio-HiFi data using minimap2–DeepVariant in all GIAB v4.2.1 regions for HG002[22]

Method	Type	Recall	Precision	F_1	TP	FN	FP
GRCh38	SNP	0.9988	0.9991	0.9990	3361165	3961	3012
CHM13-to-GRCh38	SNP	0.9988	0.9990	0.9989	3361055	4071	3482
GRCh37	SNP	0.9923	0.9979	0.9951	3326880	25805	7129
CHM13-to-GRCh37	SNP	0.9945	0.9986	0.9965	3334094	18591	4667
GRCh38	INDEL	0.9467	0.9190	0.9327	497477	27990	45387
CHM13-to-GRCh38	INDEL	0.9466	0.9189	0.9326	497426	28041	45429
GRCh37	INDEL	0.9436	0.9184	0.9308	492918	29472	45365
CHM13-to-GRCh37	INDEL	0.9448	0.9187	0.9315	493541	28849	45229

Table S10: Small variant calling accuracy for 28× WGS PacBio-HiFi data using minimap2–DeepVariant in GIAB CMRG regions for HG002[23]

Method	Type	Recall	Precision	F_1	TP	FN	FP
GRCh38	SNP	0.9876	0.9795	0.9836	17364	218	365
CHM13-to-GRCh38	SNP	0.9952	0.9890	0.9921	17497	85	196
GRCh37	SNP	0.9867	0.9698	0.9782	17668	239	553
CHM13-to-GRCh37	SNP	0.9870	0.9702	0.9785	17675	232	547
GRCh38	INDEL	0.8851	0.8700	0.8775	3204	416	496
CHM13-to-GRCh38	INDEL	0.8876	0.8760	0.8817	3213	407	470
GRCh37	INDEL	0.8816	0.8690	0.8753	3223	433	503
CHM13-to-GRCh37	INDEL	0.8829	0.8716	0.8772	3228	428	491

bioRxiv preprint doi: https://doi.org/10.1101/2022.04.27.489683; this version posted April 28, 2022. The copyright holder for this preprint (which was not certified by peer review) is the author/funder, who has granted bioRxiv a license to display the preprint in perpetuity. It is made available under aCC-BY 4.0 International license.

Table S11: Structural variant calling accuracy for 28× WGS PacBio-HiFi data using minimap2–Sniffles 2 in GRCh37 GIAB Tier 1 benchmark regions for HG002[24]

Method	Type	Recall	Precision	F_1	TP	FN	FP
GRCh37	All	0.9714	0.9238	0.9470	9365	276	772
	DEL	0.9755	0.9436	0.9593	4096	103	245
	INS	0.9682	0.9102	0.9383	5269	173	520
	Other	0	0	0	0	0	7
CHM13-to-GRCh37	All	0.9710	0.9278	0.9489	9361	280	728
	DEL	0.9740	0.9485	0.9611	4090	109	222
	INS	0.9686	0.9137	0.9403	5271	171	498
	Other	0	0	0	0	0	8

Table S12: Structural variant calling accuracy for 25× WGS PacBio-HiFi data using minimap2–Sniffles 2 in GRCh38 GIAB CMRG regions for HG002[23]

Method	Type	Recall	Precision	F_1	TP	FN	FP
GRCh38	All	0.9677	0.9519	0.9598	198	7	10
	DEL	0.9785	0.9381	0.9579	91	2	6
	INS	0.9609	0.9685	0.9647	123	5	4
CHM13-to-GRCh38	All	0.9677	0.9612	0.9644	198	7	8
	DEL	0.9785	0.9579	0.9681	91	2	4
	INS	0.9609	0.9685	0.9647	123	5	4

bioRxiv preprint doi: https://doi.org/10.1101/2022.04.27.489683; this version posted April 28, 2022. The copyright holder for this preprint (which was not certified by peer review) is the author/funder, who has granted bioRxiv a license to display the preprint in perpetuity. It is made available under aCC-BY 4.0 International license.

References

1. Nurk, S., Koren, S., Rhie, A., Rautiainen, M., Bzikadze, A. V., Mikheenko, A., Vollger, M. R., Altemose, N., Uralsky, L., Gershman, A., *et al.* The complete sequence of a human genome. *Science* **376,** 44–53 (2022).
2. Lowy-Gallego, E., Fairley, S., Zheng-Bradley, X., Ruffier, M., Clarke, L., Flicek, P., 1000 Genomes Project Consortium, *et al.* Variant calling on the GRCh38 assembly with the data from phase three of the 1000 Genomes Project. *Wellcome Open Research* **4** (2019).
3. Li, H. Aligning sequence reads, clone sequences and assembly contigs with BWA-MEM. *arXiv preprint arXiv:1303.3997* (2013).
4. Li, H. Minimap2: pairwise alignment for nucleotide sequences. *Bioinformatics* **34,** 3094–3100 (2018).
5. Jain, C., Rhie, A., Hansen, N. F., Koren, S. & Phillippy, A. M. Long-read mapping to repetitive reference sequences using Winnowmap2. *Nature Methods,* 1–6 (2022).
6. Langmead, B. & Salzberg, S. L. Fast gapped-read alignment with Bowtie 2. *Nature methods* **9,** 357 (2012).
7. Quinlan, A. R. & Hall, I. M. BEDTools: a flexible suite of utilities for comparing genomic features. *Bioinformatics* **26,** 841–842 (2010).
8. Poplin, R., Ruano-Rubio, V., DePristo, M. A., Fennell, T. J., Carneiro, M. O., Van der Auwera, G. A., Kling, D. E., Gauthier, L. D., Levy-Moonshine, A., Roazen, D., *et al.* Scaling accurate genetic variant discovery to tens of thousands of samples. *BioRxiv,* 201178 (2018).
9. McKenna, A., Hanna, M., Banks, E., Sivachenko, A., Cibulskis, K., Kernytsky, A., Garimella, K., Altshuler, D., Gabriel, S., Daly, M., *et al.* The Genome Analysis Toolkit: a MapReduce framework for analyzing next-generation DNA sequencing data. *Genome research* **20,** 1297–1303 (2010).
10. *Picard toolkit* `https://broadinstitute.github.io/picard/`. 2019.
11. Poplin, R., Chang, P.-C., Alexander, D., Schwartz, S., Colthurst, T., Ku, A., Newburger, D., Dijamco, J., Nguyen, N., Afshar, P. T., *et al.* A universal SNP and small-indel variant caller using deep neural networks. *Nature biotechnology* **36,** 983 (2018).
12. Krusche, P., Trigg, L., Boutros, P. C., Mason, C. E., Francisco, M., Moore, B. L., Gonzalez-Porta, M., Eberle, M. A., Tezak, Z., Lababidi, S., *et al.* Best practices for benchmarking germline small-variant calls in human genomes. *Nature biotechnology* **37,** 555–560 (2019).
13. Smolka, M., Paulin, L. F., Grochowski, C. M., Mahmoud, M., Behera, S., Gandhi, M., Hong, K., Pehlivan, D., Scholz, S. W., Carvalho, C. M., *et al.* Comprehensive Structural Variant Detection: From Mosaic to Population-Level. *bioRxiv* (2022).

bioRxiv preprint doi: https://doi.org/10.1101/2022.04.27.489683; this version posted April 28, 2022. The copyright holder for this preprint (which was not certified by peer review) is the author/funder, who has granted bioRxiv a license to display the preprint in perpetuity. It is made available under aCC-BY 4.0 International license.

14. Martin, M., Patterson, M., Garg, S., Fischer, S. O., Pisanti, N., Klau, G. W., Schöenhuth, A. & Marschall, T. WhatsHap: fast and accurate read-based phasing. *BioRxiv,* 085050 (2016).
15. English, A. C., Menon, V. K., Gibbs, R., Metcalf, G. A. & Sedlazeck, F. J. Truvari: Refined Structural Variant Comparison Preserves Allelic Diversity. *bioRxiv* (2022).
16. Talenti, A. & Prendergast, J. nf-LO: A Scalable, Containerized Workflow for Genome-to-Genome Lift Over. *Genome Biology and Evolution* **13,** evab183 (2021).
17. Fujita, P. A., Rhead, B., Zweig, A. S., Hinrichs, A. S., Karolchik, D., Cline, M. S., Goldman, M., Barber, G. P., Clawson, H., Coelho, A., *et al.* The UCSC genome browser database: update 2011. *Nucleic acids research* **39,** D876–D882 (2010).
18. Zhao, H., Sun, Z., Wang, J., Huang, H., Kocher, J.-P. & Wang, L. CrossMap: a versatile tool for coordinate conversion between genome assemblies. *Bioinformatics* **30,** 1006–1007 (2014).
19. Holtgrewe, M. Mason: a read simulator for second generation sequencing data. *Technical Reports of Institut für Mathematik und Informatik, Freie Universität Berlin* **TR-B-10-06** (2010).
20. Gordon, A. *GNU Time* `https://www.gnu.org/software/time/`. 2018.
21. Thorvaldsdóttir, H., Robinson, J. T. & Mesirov, J. P. Integrative Genomics Viewer (IGV): high-performance genomics data visualization and exploration. *Briefings in bioinformatics* **14,** 178–192 (2013).
22. Wagner, J., Olson, N. D., Harris, L., Khan, Z., Farek, J., Mahmoud, M., Stankovic, A., Kovacevic, V., Wenger, A. M., Rowell, W. J., *et al.* Benchmarking challenging small variants with linked and long reads. *BioRxiv* (2020).
23. Wagner, J., Olson, N. D., Harris, L., McDaniel, J., Cheng, H., Fungtammasan, A., Hwang, Y.-C., Gupta, R., Wenger, A. M., Rowell, W. J., *et al.* Curated variation benchmarks for challenging medically relevant autosomal genes. *Nature Biotechnology,* 1–9 (2022).
24. Zook, J. M., Hansen, N. F., Olson, N. D., Chapman, L., Mullikin, J. C., Xiao, C., Sherry, S., Koren, S., Phillippy, A. M., Boutros, P. C., *et al.* A robust benchmark for detection of germline large deletions and insertions. *Nature biotechnology* **38,** 1347–1355 (2020).
